# From roadmap to action: A pathway toward enhanced resilience in the U.S. beef value chain

**DOI:** 10.1093/af/vfag037

**Published:** 2026-07-07

**Authors:** Samantha J Werth, Reba L Colin, Douglas J Goodwin, Hugh Aljoe

**Affiliations:** U.S. Roundtable for Sustainable Beef, Centennial, CO; National Cattlemen’s Beef Association, Centennial, CO; Center for Grazinglands and Ranch Management, Texas A&M University, College Station, TX; Noble Research Institute, Ardmore, OK

**Keywords:** collaboration, data interoperability, decision-support tools, funding mechanisms, sustainability indicators

## Abstract

**Graphical Abstract:**
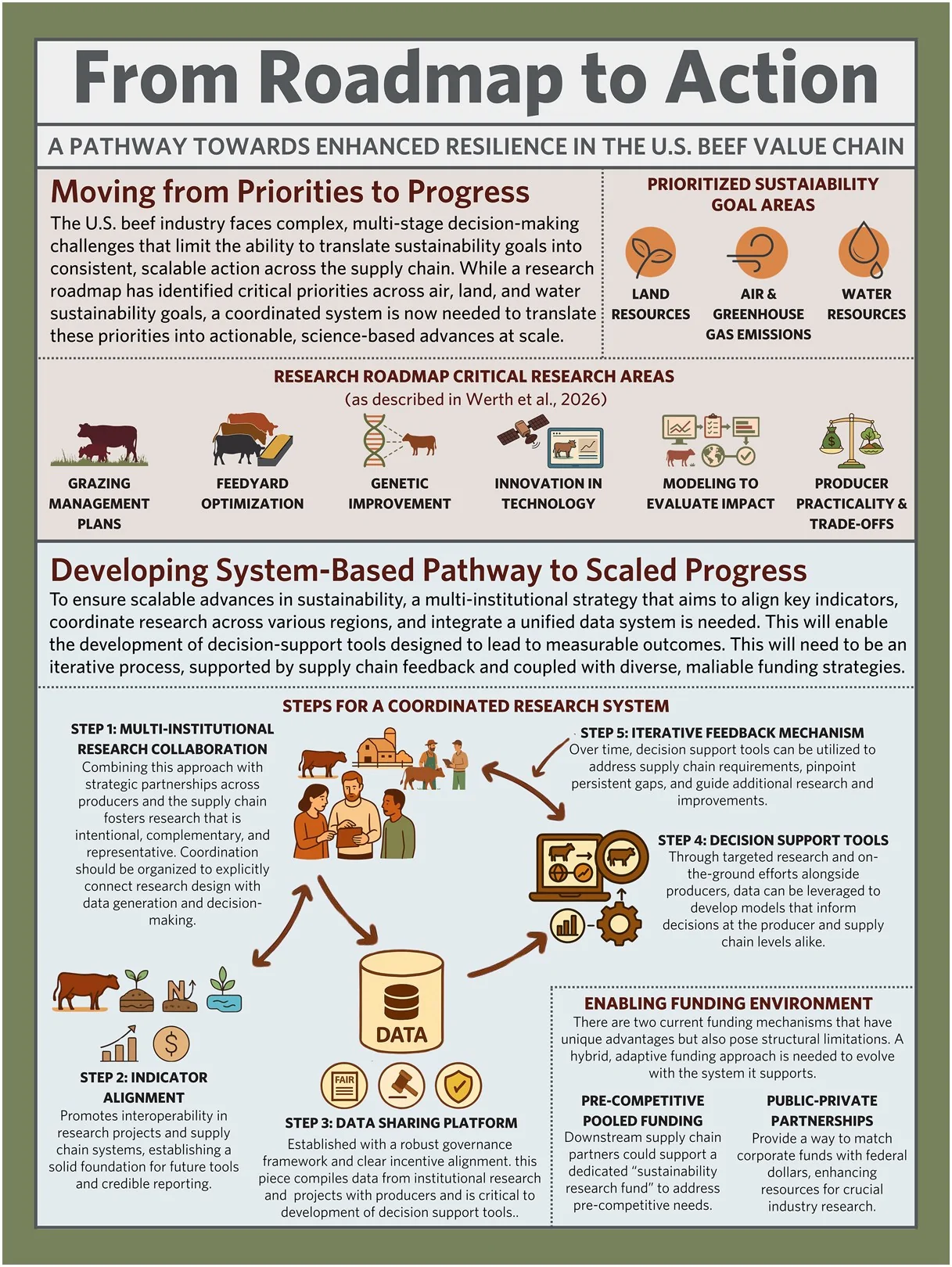

Implications

- A coordinated, cross‑sector research collaborative is essential to translate sustainability priorities into scalable, real‑world outcomes across the U.S. beef value chain.
- Early alignment on credible, feasible sustainability indicators enables interoperability across institutions, supports consistent measurement, and strengthens supply‑chain reporting.
- Establishing a trusted, secure data‑sharing platform will accelerate model development and improve decision‑making for producers and downstream stakeholders.
- Multi-institutional, multi‑regional research—paired with iterative, producer-first feedback loops—ensures that tools and insights remain relevant, practical, and adaptable.
- Pre‑competitive pooled funding or public–private partnership structures provide the financial architecture needed to sustain long-term research progress and industry-wide resilience.

## Introduction: Moving from Priorities to Progress

The decision-making process in the U.S. cattle industry is complex, spanning multiple sectors across the beef life cycle and shaped by market fluctuations, resource availability, and regional differences (Lai et al., 2026). At the producer level, key decision-making considerations include weather, herd size, operational goals, and financial drivers. Although these complexities are outlined in more detail in the companion paper by Lai et al. (2026), recognizing them is essential for developing strategies that reflect producer realities while advancing environmental, social, and economic goals across the supply chain.

In 2024, the U.S. Roundtable for Sustainable Beef (USRSB) convened stakeholders across the supply chain to understand the current state of the science and to identify critical gaps in the science around their industry-wide sustainability goal areas of Air & Greenhouse Gas Emissions, Land Resources, and Water Resources (USRSB, 2022). This effort resulted in the development and publication of a targeted research roadmap in the companion article by Werth et al. (2026). This roadmap outlined the specific research needs across the goal areas of Air & Greenhouse Gas Emissions, Land Resources, and Water Resources in order to advance sustainability in U.S. beef production.

To translate the priorities outlined in Werth et al. (2026) from roadmap to action, a systematic approach is required. This approach must rely on collaborative efforts across research institutions, alignment on data-sharing protocols, and access to both traditional and non-traditional funding sources. Addressing the various operational types and landscapes across regions will require researchers to conduct multi-year, cross-disciplinary, multi-institutional, and multi-regional experiments to provide the most relevant and effective insights for the industry and its stakeholders. These factors make it imperative that researchers have better and more consistent access to both public and private funds and the opportunity to partner with researchers across other universities, the federal government, and the supply chain. It will also be imperative to have producers at the table, not just to help inform research needs, but to play an active role in designing viable projects and leading on-the-ground efforts alongside researchers and beyond controlled research environments. Although ambitious, successful implementation of the roadmap provides an opportunity for advancing industry ambitions on sustainability. This perspective piece therefore advances a proposed implementation architecture that links indicator alignment, coordinated research, interoperable data systems, and decision-support tools to translate the research roadmap into scalable, supply-chain-relevant action (Figure 1).

**Figure 1.**
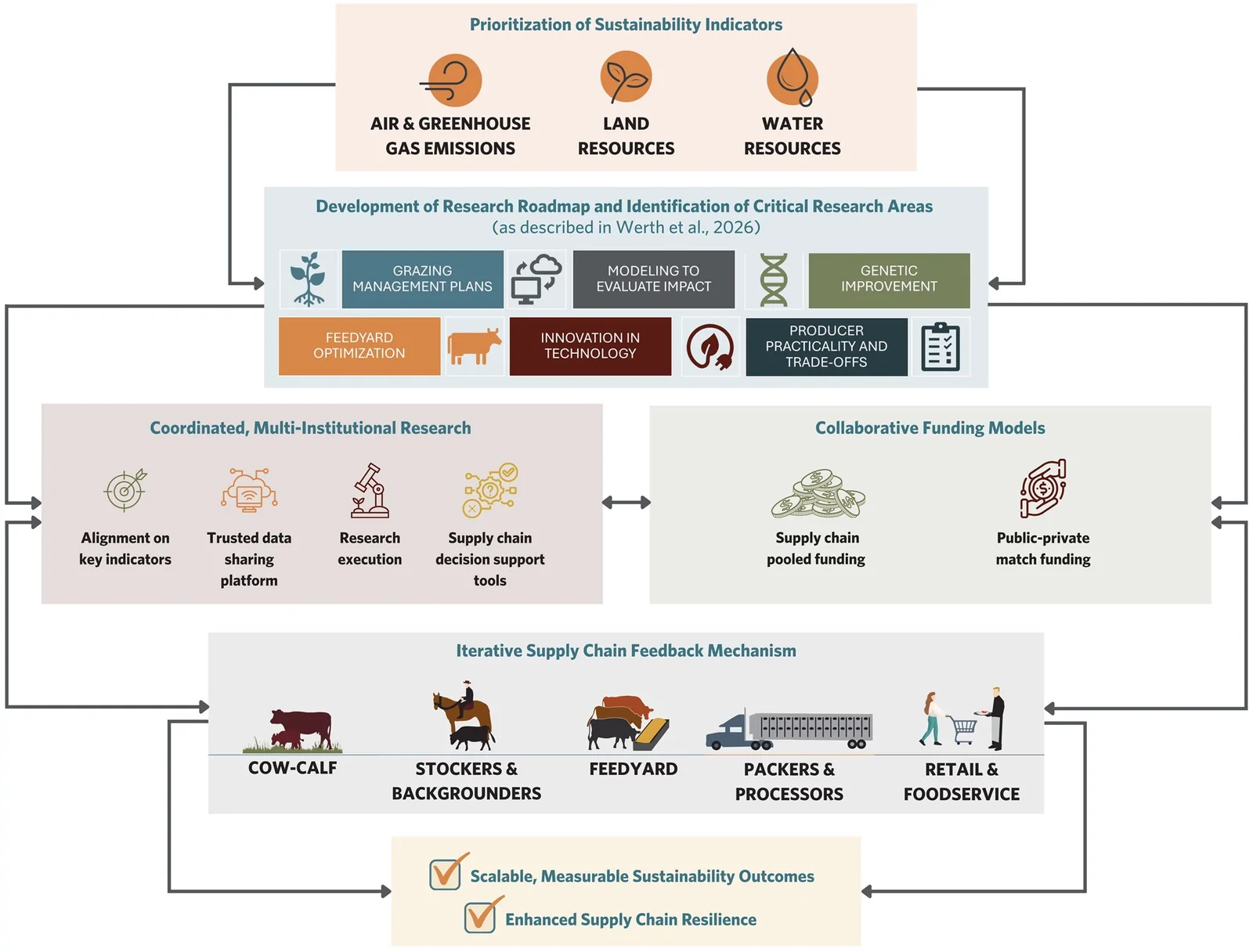
Moving from roadmap to action: beginning with prioritization of sustainability indicators and the development of the guiding research roadmap (Werth et al., 2026), progressing to coordinated, multi-institutional research catalyzed by collaborative funding models and informed through an iterative supply chain feedback mechanism, and finally leading to scalable, measurable sustainability outcomes and enhanced supply chain resilience.

## The Need for a Coordinated, Cross-sector Collaborative

Translating sustainability priorities into measurable outcomes requires not only collaboration, but a coordinated system in which research, data, and decision-making are explicitly linked. To achieve long-term, sustained advances in sustainability for the beef industry, it is essential to move beyond short-term fixes and focus on systemic shifts that build resilience and adaptability across the industry. Historically fragmented research efforts slow progress, while methods, data, and insights often fail to translate across institutions or into producer operations. Regional differences have often led to a lack of coordinated efforts and have kept research and corresponding insights confined to specific location and institution. Meanwhile, producers, packers and processors, retail and foodservice companies, investors, and policymakers increasingly require consistent indicators, credible measurement, and actionable tools.

Werth et al. (2026) emphasized that no individual institution or sector can address these challenges alone. Coordinated approaches increase statistical power through data aggregation, improve comparability through standardized methodologies, and enhance the relevance of findings by incorporating diverse production systems (Tsegaye et al., 2024). For successful progress, the industry requires more clear and consistent data and insights across various regions and operational types. To achieve this, coordination must be structured in a way that explicitly links research design, data generation, and decision-making.

Similar coordination challenges in agricultural and environmental systems have been addressed through pre-competitive, multi-stakeholder collaboratives that enable standardization of metrics, pooling of data, and accelerated translation of research into practice. The USDA Long-Term Agroecosystem Research (LTAR) network has demonstrated the value of coordinated, long-term, cross-site research designed to improve agricultural productivity while sustaining ecosystem services (Tsegaye et al., 2024; USDA, 2026). Similarly, the FAO Livestock Environmental Assessment and Performance (LEAP) Partnership has established globally recognized methodologies for assessing environmental performance across livestock supply chains through multi-stakeholder consensus (FAO, 2024). In this context, cross-sector collaboration is not simply aspirational, but it is a mechanism to overcome structural barriers to scale by aligning research design, enabling interoperable data systems, and linking scientific outputs directly to supply chain decision-making.

## Taking the First Steps Toward Coordinated Research and Action

The proposed pathway to implementation can be understood as a connected system. Indicator alignment enables interoperable data, which in turn supports coordinated research and ultimately the development of decision-support tools that inform action across the supply chain (Figure 1). This system will require a phased implementation approach, as proposed below:

- Phase 1 (0 to 2 years): indicator alignment, governance development, and development of data-sharing systems
- Phase 2 (2 to 5 years): expansion to regional research networks and integration of producer-led trials
- Phase 3 (5+ years): deployment of interoperable data systems and supply-chain-integrated decision tools.

This phased approach mirrors successful implementation models observed in coordinated research networks such as LTAR, where common measurements and cross-site experiments are scaled over time to generate nationally relevant insights (USDA, 2026). However, such an approach is not without its challenges, and to achieve successful implementation of the roadmap, these challenges must be addressed.

First and foremost, alignment on key indicators is a prerequisite across air, land, and water to address the most pressing research gaps identified in the roadmap (Figure 1). Initial alignment can focus on a limited set of high-priority indicators already identified through the roadmap process, including enteric methane emissions, soil carbon dynamics, nitrogen losses to water systems, and productivity- or efficiency-related metrics that link environmental performance with economic outcomes (Werth et al., 2026). Indicators must be scientifically rigorous, operationally feasible, and compatible with supply chain reporting needs, including scope 3 emissions accounting, while remaining adaptable across diverse regions and production systems. Early alignment on indicators ensures interoperability across research projects, research institutions, and supply chain systems while building a sound foundation for future tools and reporting credibility.

In parallel, a data-sharing platform must be established to ensure industry-wide understanding and representation. Beyond interoperability, data governance structures must define ownership, access, and use rights. Adopting principles aligned with FAIR data (Findable, Accessible, Interoperable, Reusable) provides a foundation for ensuring consistency and usability across datasets (Wilkinson et al., 2016). Furthermore, tiered-access models can enable aggregated, anonymized producer data to inform system-level analyses while protecting individual operations. Importantly, this shifts data from being an output of individual studies to a shared resource that supports cumulative learning across the system.

The willingness of institutions, producers, and other supply chain partners to share data and create alignment across research is a challenge that does not lend itself a simple solution. Tensions around intellectual property and data ownership can restrict collaboration, particularly when proprietary technologies or datasets are involved. This is where an emphasis on fairness and trust across the supply chain along with clear data governance is essential. Furthermore, a sound mechanism by which producers can be fairly compensated for their time and data provided, regardless of a desired project outcome, must be foundational to a data-sharing platform. A secure data-sharing platform, established under a strong governance framework, with clear incentive alignment, that houses key data from institutional research as well as on-the-ground projects with producers, can then be leveraged to support development of powerful decision-support tools (i.e. models) for each stage of the supply chain (Figure 1).

With alignment on indicators and a trusted data-sharing platform, addressing the critical research areas from the research roadmap becomes more feasible, enabling a collaborative and outcome-based approach for researchers and supply chain partners alike. Research must be rooted in real operational constraints while being aware of market needs and regional variabilities. Additionally, a multi-institutional approach combined with a transparent and iterative learning process that bridges upstream producers and downstream supply chain partners is essential to ensuring that research remains relevant and scalable.

To support a transparent and iterative process, the final step to successfully implementing the research roadmap is the development of sector-specific decision-support tools (Figure 1). Through targeted research and on-the-ground efforts alongside producers, data can be leveraged to develop models that inform decisions at the producer and supply chain levels alike. Grounded in a shared goal of scalable solutions that strengthen supply chain resilience, over time these decision-support tools can be utilized to ensure scalable and long-term progress.

## Funding Mechanisms to Support Scalable Progress

There are two main pathways by which a large-scale, multi-institutional research collaborative may be funded: pre-competitive pooled funding or blended financing through public-private partnership (Figure 1). Either of these approaches would support advancing the next steps of tackling the research gaps identified in Werth et al. (2026), although both present challenges that must be overcome to ensure long-term success.

With uncertainties in federal funding mechanisms in the United States, the need for strategic partnerships and industry support is more critical than ever. A pre-competitive pooled funding approach is one mechanism that would ensure consistent funding for the industry’s most critical needs without reliance on government support. There are several examples of successful programs already in place across the United States through agricultural check-off programs, such as the Beef Check-Off, in which funds are collected from the sale of commodities and then pooled to support a variety of topics important to the respective commodity groups. Although these programs are broader in their scope, downstream supply chain partners could adopt a similar approach by developing a dedicated “sustainability research fund” to address pre-competitive needs. Unlike commodity check-off programs, which rely on mandated assessments, this sort of proposed pooled funding mechanism would depend on voluntary participation across supply chain partners. Although this introduces additional coordination challenges, a voluntary fund could be established under strong governance and multi-stakeholder guidance to ensure that funds are utilized to address the industry’s most pressing sustainability needs and distributed in an objective manner via a request for proposals process, similar to how check-off research is currently set up. This sort of approach would ensure long-term, consistent funding for advances in sustainability.

Public-private partnerships have long been a successful means of supporting large-scale research efforts that tackle industry challenges. The Foundation for Food & Agriculture Research (FFAR) in the United States, CSIRO in Australia, and AgriZeroNZ in New Zealand are all examples of institutions that have built sound foundations in public-private partnerships, and their successes are evidence of the power of such partnerships (CSIRO, 2025; FFAR, 2025; AgriZeroNZ, 2026). These programs provide a means of matching funds from corporations, thus helping to extend the impact of resources contributed to these industry-important research needs. Public–private consortia supported by FFAR, such as the Metrics, Management, and Monitoring (3M) project and the International Consortium for Antimicrobial Stewardship in Agriculture (ICASA), have demonstrated the ability to mobilize multi-institutional collaboration and matched funding to address targeted research challenges (FFAR, 2025, 2026; Noble Research Institute, 2026). Corporate- and NGO-led pilot programs, including projects supported through the National Fish and Wildlife Foundation (NFWF) and initiatives such as the Legacy Landscapes program, further demonstrate growing investment in producer-facing sustainability interventions, though these efforts remain largely localized and non-interoperable (NatGLC, 2026; NFWF, 2026).

Collectively, these examples highlight that there is no single, proven financing model capable of supporting a collaborative effort of this scale across the U.S. beef value chain. While pooled funding mechanisms and public–private partnerships each offer distinct advantages, they also carry structural limitations that may constrain their ability to operate at the required level. As such, the path forward will likely require a hybrid, adaptive funding approach that evolves alongside the system it is designed to support.

Ultimately, the success of this effort will depend not on identifying a perfect funding mechanism at the outset, but on building a shared commitment across the supply chain to co-invest in pre-competitive research infrastructure. This will require thinking beyond traditional funding paradigms and developing new partnership models that align incentives, distribute risk, and ensure sustained investment in science-based solutions. In this way, funding should be viewed not only as a resource constraint, but as a design opportunity to create durable, collaborative systems capable of supporting long-term sustainability progress across the industry.

## Conclusion: A Unified Path Toward Measurable Progress

The research roadmap has clarified the work that must be done and the priorities that must be addressed for progress across the beef value chain (Werth et al., 2026); the industry now needs clarity on how to accomplish this, and the work presented herein provides a place from which to build. The framework presented here moves beyond prioritization to propose an integrated system for implementation at scale. A coordinated, multi-institutional, and cross-sector research collaborative, funded through pre-competitive means, creates the execution architecture needed to translate research priorities into real, scalable solutions. By aligning credible indicators, standing up a secure data-sharing platform, and building producer-first decision-support tools, the beef value chain can deliver measurable improvements in air, land, and water across diverse regions while strengthening economic resilience and social understanding. This approach must be iterative and flexible so that the industry is able to adapt as evidence evolves, markets shift, and technology advances. With shared principles, clear milestones, and sustained co-investment, the U.S. beef sector can move from scattered efforts to durable, scalable progress, turning research into action that benefits producers, communities, and the environment while ensuring resilience of the U.S. beef supply chain.

## Author Biographies

**Dr. Samantha J. Werth** serves as Executive Director with the Global Enteric Methane Impact Alliance (GEMIA). In this role, Sam works collaboratively alongside a variety of stakeholders to support advancement of applied solutions to reducing emissions from livestock production. Prior to her role with GEMIA, Sam served as executive director of the U.S. Roundtable for Sustainable Beef (USRSB) where she focused on building collaborative partnerships to support USRSB’s sustainability initiatives and supported members with their sustainability efforts. Sam is a native of California and completed her undergraduate, master’s, and doctoral studies at the University of California, Davis. Her graduate studies centered around ruminant nutrition and sustainable beef production. Sam is passionate about leveraging science-based information to support advances in sustainable beef production.

**Reba L. Colin**, MS, is the Associate Director of Sustainability Projects at the National Cattlemen’s Beef Association. In this role, she supports producers in elevating their sustainability story along with developing educational materials and events related to key sustainability topics. She holds a Master of Science degree in Ruminant Nutrition from the University of Nebraska-Lincoln and a Bachelor of Science degree in Animal Science from Kansas State University. She grew up on a small cow/calf operation in West Central Missouri, which instilled a love for the beef industry and supporting fellow cattle producers.

**Dr. Douglas J. Goodwin** serves as Research Associate Professor for Texas A&M AgriLife Research and Extension and as Director of the Center for Grazinglands and Ranch Management within the Department of Rangeland, Wildlife, and Fisheries Management. The Center focuses on providing applied, ranch-relevant research and education, prioritizing the ecological and economic resiliency of grazingland resources and ranching operations. Prior to Texas A&M, Dr. Goodwin served as Conservation Stewardship Lead and Senior Rangeland and Pasture Consultant for Noble Research Institute in Ardmore, Oklahoma. Dr. Goodwin has over 24 years of experience working directly with ranchers and grazingland managers, implementing stewardship-focused management and providing innovative solutions to grazingland issues.

**Hugh Aljoe** serves as the director of ranches, outreach, and partnerships at Noble Research Institute, where he focuses on regenerative management of range and pasture, high-stock-density grazing, and holistic ranch management. He has been associated with Noble since 1995 and, before joining, managed a 3,000-acre, 1,500-head cattle operation in Texas. Hugh received his master’s degree in range science from Texas A&M University with an emphasis on grazing management.

## References

[vfag037-B1] AgriZeroNZ. 2026. AgriZeroNZ. Powering zero emissions agriculture. Available from: https://www.agrizero.nz/

[vfag037-B2] CSIRO. 2025. CSIRO annual report 2024-2025. Canberra, Australian Capital Territory. Available from: https://www.csiro.au/annualreport2025

[vfag037-B3] FAO. 2024. FAO livestock environmental assessment and performance partnership. Rome. Available from: https://www.fao.org/partnerships/leap

[vfag037-B4] FFAR. 2025. A decade of advancing bold food & agriculture research. Available from: https://foundationfar.org/wp-content/uploads/2025/05/FFAR2024ImpactReport-FINAL-29May25.pdf

[vfag037-B5] FFAR. 2026. International Consortium for Antimicrobial Stewardship in Agriculture (ICASA): advancing antimicrobial stewardship research in animal agriculture. Foundation for Food & Agriculture Research. Available from: https://foundationfar.org/consortia/international-consortium-for-antimicrobial-stewardship-in-agriculture/

[vfag037-B6] Lai E. , Klopatek S. C., Werth S. J. 2026. The case for a sustainable beef research roadmap: building consensus on beef sustainability. Anim. Front.

[vfag037-B7] NatGLC. 2026. Legacy landscapes grazing lands program. National Grazing Lands Coalition. Available from: https://www.grazinglands.org/legacy-landscapes

[vfag037-B8] NFWF. 2026. NFWF programs. National Fish and Wildlife Foundation. Available from: https://www.nfwf.org/programs

[vfag037-B9] Noble Research Institute. 2026. Metrics, management, & monitoring (3M): an investigation of pasture and rangeland soil health and its drivers. Noble Research Institute. Available from: https://www.noble.org/research/3m/

[vfag037-B10] Tsegaye T. , Eve M., Hapeman C. J., Kleinman P. J. A., Baffaut C., Browning D. M., Coffin A. W., Spiegal S. A. 2024. The long-term agroecosystem research (LTAR) network: cross-site transdisciplinary science to support a sustainable and resilient agriculture. J. Environ. Qual. 53:777–786. 10.1002/jeq2.20649 39500565

[vfag037-B11] USDA. 2026. The long-term agroecosystem research (LTAR) network. Available from: https://ltar.ars.usda.gov/10.1002/jeq2.70027 PMC12431957 40281665

[vfag037-B12] USRSB. 2022. USRSB high-priority indicator goals & sector targets. Available from: https://www.usrsb.org/our-work/our-goals

[vfag037-B13] Werth S. J. , Vehige G. D., Place S. E., Thompson L. R., Rowan T. N., Goodwin D. J., Rowntree J. E., Stanley P. L., Metzger S. T., Wagner K. L. 2026. Innovation in the U.S. beef value chain: a sustainable beef research roadmap. Anim. Front.

[vfag037-B14] Wilkinson M. D. , Dumontier M., Aalbersberg J., Appleton G., Axton M., Baak A., Blomberg N., Boiten J. W., da Silva Santos L. B., Bourne P. E. et al 2016. Comment: the FAIR guiding principles for scientific data management and stewardship. Sci. Data. 3:160018. 10.1038/sdata.2016.18 PMC4792175 26978244

